# The distribution of D4Z4 repeats in China and direct prenatal diagnosis of FSHD by optical genome mapping

**DOI:** 10.1186/s13023-025-03591-w

**Published:** 2025-02-11

**Authors:** Mengmeng Li, Na Hao, Jiazhen Chang, Kaili Yin, Xueting Yang, Yaru Wang, Yi Dai, Yulin Jiang

**Affiliations:** 1 Department of Obstetrics, Peking Union Medical College Hospital, National Clinical Research Center for Obstetric & Gynecologic Diseases, Chinese Academy of Medical Science and Peking Union Medical College, No.1 Shuaifuyuan, Dongcheng District, Beijing, 100730 China; 2 Ecobono (Beijing) Biotech Co., Ltd, Beijing, China; 3 Department of Neurology, Peking Union Medical College Hospital, Chinese Academy of Medical Science and Peking Union Medical College, Beijing, 100730 China

**Keywords:** FSHD, Prenatal diagnosis, Optical genome mapping

## Abstract

**Background:**

Facioscapulohumeral muscular dystrophy (FSHD) is the second most common form of muscular dystrophy, which is characterized by a reduction in the number of D4Z4 repeats on chromosome 4q35. Prenatal diagnosis of FSHD has been challenging due to the large quantity and high-quality DNA required for Southern blot (SB) analysis. Optical genome mapping (OGM) technology has shown promise in identifying repeat contraction disorders and presents a potential tool for the prenatal diagnosis of FSHD.

**Methods:**

In this retrospective cohort study, we investigated the distribution of D4Z4 repeats in 100 unrelated healthy individuals from the Chinese Han population using peripheral blood samples and DLS labelling method. Additionally, prenatal diagnosis using OGM was performed in 12 FSHD families at Peking Union Medical College Hospital between January 2021 and December 2023. The prenatal samples included 2 amniotic cell cultures and 10 chorionic villus samples (CVS), with 9 labeled using DLS and 4 using NLRS method.

**Results:**

Among the 100 healthy controls, the distribution of D4Z4 repeats varied, with 3 individuals having borderline 10 repeat counts on 4qA, and the most frequent count being 14 units. One individual with mosaicism was also identified. In the cohort of 12 FSHD families,14 prenatal diagnoses were performed. Of these 14 cases, 4 fetuses tested positive for 4qA contraction, with repeat counts ranging from 2 to 4. In both families that underwent two rounds of prenatal diagnosis, the first diagnosis indicated the presence of FSHD, leading to pregnancy termination, while the second diagnosis confirmed the presence of healthy fetuses. The overall positive rate was 28.57%.

**Conclusion:**

Our findings demonstrate that OGM is an accurate and effective method for the prenatal diagnosis of FSHD. The application of OGM in prenatal settings could offer significant benefits to families affected by FSHD with reproductive concerns.

## Introduction

Facioscapulohumeral muscular dystrophy (FSHD) is a genetically complex, autosomal dominant inheritance disorder and one of the most common inherited muscular dystrophies [1]. FSHD is characterized by progressive weakness of the facial, scapular and humeral muscles, often presenting asymmetrically [2]. The estimated prevalence of FSHD is estimated at 1/20,000, with complete penetrance by age 20 [3]. Clinically, FSHD is heterogeneous and has been subdivided into two forms: FSHD1 and FSHD2 [4]. FSHD1, which accounts for 95% of all FSHD cases, is associated with the contraction of the D4Z4 macrosatellite repeat region on chromosome 4q35 [5]. Specifically, FSHD1 patients exhibit a reduced number of D4Z4 repeats (typically fewer than 10) on the permissive 4qA allele [6].

Traditionally, prenatal diagnosis of FSHD has relied on Southern blot (SB) analysis to detect the size of the D4Z4 repeat [7]. Although SB has long been a gold-standard technique, it presents several challenges in the context of prenatal diagnosis. It is labor-intensive, requires a large amount of high-quality DNA, and is time consuming, making it problematic in time-sensitive prenatal settings [8]. Therefore, while prenatal diagnosis of FSHD using SB presents significant challenges in clinical practice, it has become a pressing necessity for families with a history of the disease. Additionally, the distribution of D4Z4 repeat numbers in various populations, particularly in the healthy Chinese Han population, remains poorly understood.

With the advent of optical genome mapping (OGM), significant advantages have emerged for FSHD diagnosis, particularly in prenatal settings. OGM offers a promising alternative to traditional methods [9]. This high-resolution technology can sensitively and accurately detect D4Z4 repeats, distinguishing between the permissive 4qA allele and non-permissive 4qB allele [10]. OGM also excels at identifying complex genomic rearrangements and mosaicism, which may be missed by SB [11]. Furthermore, OGM`s shorter turnaround time makes ita more efficient and precise tool for prenatal diagnosis of FSHD.

In this study, we utilized OGM to investigate the distribution of D4Z4 repeats in a cohort of healthy individuals from the Chinese Han population and performed direct prenatal diagnosis of FSHD in families with an urgent need. The findings will contribute to a deeper understanding of D4Z4 repeat patterns and offer valuable insights into prenatal diagnostic practices for FSHD.

## Methods

### Cohort sample

The control group comprised 100 unrelated healthy individuals with no family history of FSHD, recruited from Peking Union Medical College Hospital.

**Fig. 1:**
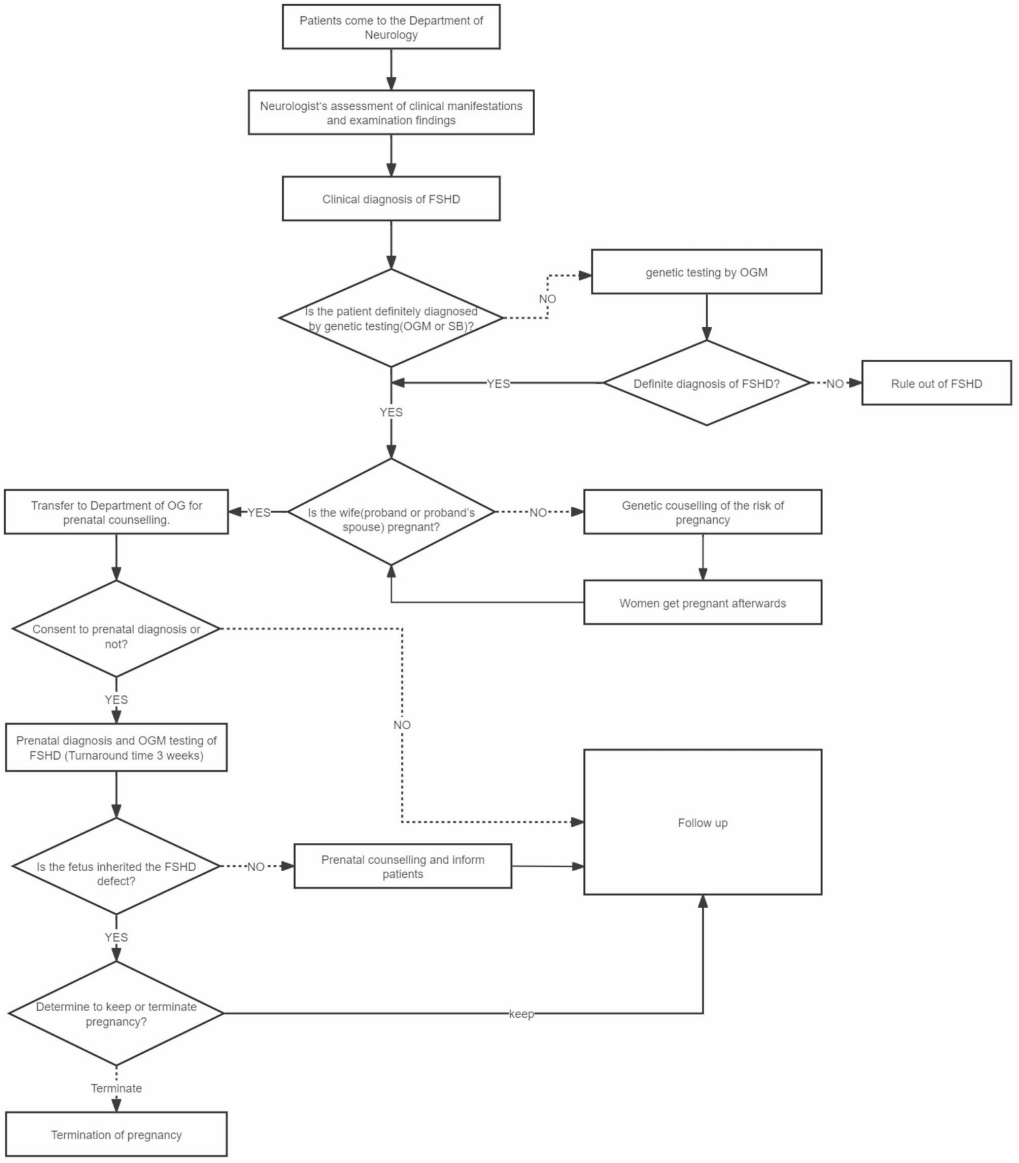
Flow chart of prenatal diagnosis for FSHD

Between January 2021 and December 2023, we retrospectively enrolled 12 families who sought genetic counseling and prenatal diagnosis for FSHD. A detailed enrollment flowchart outlining participant selection is provided in Fig. 1. The prenatal diagnosis of FSHD in these families was based on three types of samples: peripheral blood from all 12 families, chorionic villus tissue from 12 cases, and amniotic fluid from 2 cases. This study was approved by the Ethics Committee of Peking Union Medical College Hospital (Reference number: I-22PJ1063). All enrolled subjects have consented to participate.

### Sample processing

#### DNA extraction and quantification

DNA was extracted from three different sources following specific protocols. For amniotic fluid cells, 10 mL of cells was cultured to 800,000 cells. The extraction process involved cell lysis with proteinaseK, isopropanol precipitation of DNA, and nanodisk adsorption of ultra-high molecular weight (UHMW) DNA, followed by washing and homogenization, according to the Bionano Prep SP Amnio and CVS Culture DNA Isolation Protocol. Peripheral blood UHMW DNA was obtained by thawing frozen peripheral blood and using 1.5 million leukocytes. The extraction procedure was based on the Bionano Prep SP Frozen Human Blood DNA Isolation Protocol v2. Chorionic tissue extraction was performed using 10 mg of tissue, involving tissue homogenization, cell lysis, isopropanol precipitation of DNA, nanodisk adsorption of UHMW DNA, as described in the Bionano SP Tissue and Tumor DNA Isolation Protocol. Extracted genomic DNA was quantified using a Qubit dsDNA BR assay kit and a Qubit 4.0 fluorometer (Thermo Fisher Scientific).

#### DNA labeling

Two different labeling protocols were used for DNA labeling: Direct Label and Stain (DLS) and Nick Label Repair Stain (NLRS). For the 109 samples labeled using DLS, 750 ng of gDNA was labelled with DLE-1 enzyme at a specific CTTAAG motif. The DNA backbone was then stained blue. The concentration of labeled DNA was maintained between 4 and 12 ng/µl. For NLRS, 300 ng of gDNA was nicked at the single molecule level using the endonuclease Nb.BssSI in 5 samples. Labeling was performed by a limited-drive nick translation process in the presence of a fluorophore-labeled nucleotide. The labeled nicks were repaired to restore strand integrity. The labelled DNA was stained for backbone visualization.

### Data analysis

DLS data analysis was conducted using the EnFocus™ FSHD Pipeline. This process involved performing a local assembly of regions of interest, selecting molecules aligned to these regions and assembling them. The resulting genomic maps in the chr4 and chr10 D4Z4 regions were analyzed to size the repeats and assign haplotypes to the alleles. Data quality criteria included an N50 value of ≥ 200 kbp, a mapping rate of ≥ 70%, an average label density of 14–17 labels per 100 kbp, an effective coverage of ≥ 75× across the whole genome, a positive label variance of 3–10%, and a negative label variance of 6–15%.

NLRS data analysis focused on the D4Z4 target region of the whole genome map. The number of D4Z4 repeats was determined by calculating the number of restriction sites in the target region. The presence of an additional Nb.BssSI site in the 4qA haplotype was used to distinguish between the 4qA/4qB and 10qA/10qB haplotypes.

## Results

### The distribution of D4Z4 in Chinese Han population

We investigated 100 unrelated healthy individuals with no family history of FSHD from the Chinese Han population using OGM. Among these individuals, 59.00% were female (59/100), and 41.00% were male (41/100), with an average age of 31.91 ± 5.08 years. The frequency of 4qA homozygosity is 13.00% (13/100), with 46.15% in males (6/13) and 53.85% in females (7/13). The frequency of 4qB homozygosity is 35.00% (35/100), with 42.86% in males (15/35) and 57.14% in females (20/35). Additionally, the frequency of 4qA/4qB heterozygosity is 52.00% (52/100), with 38.46% in males (20/52) and 61.54% in females (32/52). The gene frequency of 4qA is 38.80% and that of 4qB is 61.20%.

**Fig. 2:**
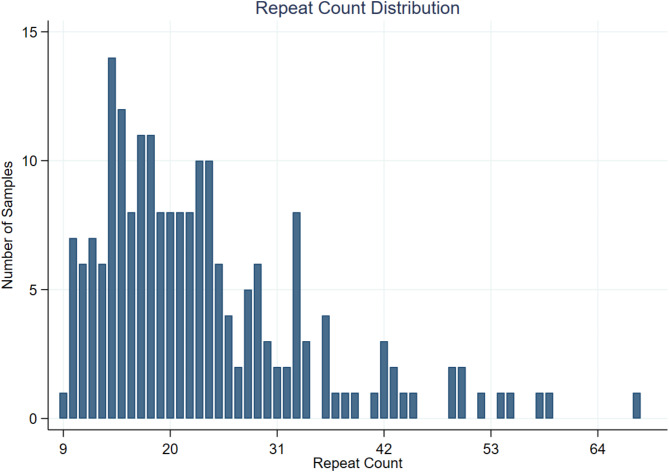
The distribution of D4Z4 repeat counts among the 100 controls

Figure 2 illustrates the distribution of D4Z4 repeat counts among the 100 controls. Three individuals exhibited 10-unit repeats on 4qA allele, which is considered borderline for FSHD. 14% of the control samples had 9–11 units. The most frequent repeat count observed was 14 units, occuring 14 times. Notably, one individual with mosaicism was identified, carrying a mixture of two 4qB allele and one 4qA allele.

### Direct prenatal diagnosis of FSHD in 12 FSHD families

We included 12 FSHD families, all in urgent need of prenatal diagnosis, were included. The clinical information of the 12 probands is summarized in Table 1, with all probands presenting the classical FSHD phenotypes. The median age at onset for the probands was 19.00 years (interquartile range:13.50–22.00 years). The D4Z4 repeat counts ranged from 2 to 5.

**Table 1:** Clinical data of the facioscapulohumeral muscular dystrophy (FSHD) probands

FSHD families	Subject Sex	Age (years)	D4Z4 repeat count		Age at onset (years)	Neurological examination
FSHD 1	female	31	2	4qA	13	typical facial weakness, scapular winging, arm abduction below shoulder level, lumbar lordosis, presence of Beevor’s sign.
			19	4qA		
FSHD 2	male	30	3	4qA	20	typical facial weakness, scapular winging, proximal weakness, weakness and atrophy of biceps asymmetrically, presence of Beevor’s sign.
			49	4qA		
FSHD 3	female	29	4	4qA	20	weakness of orbicularis oris muscle, scapular winging, weakness and atrophy of biceps, foot dorsiflexion weakness.
			30	4qB		
FSHD 4	male	36	3	4qA	10	typical facial weakness, scapular winging, arm abduction below shoulder level, lumbar lordosis, presence of Beevor’s sign, foot dorsiflexion weakness.
			23	4qB		
FSHD 5	male	37	5	4qA	24	scapular winging, proximal weakness, lumbar lordosis, presence of Beevor’s sign.
			31	4qA		
FSHD 6	male	29	4	4qA	19	weakness of orbicularis oris muscle, scapular winging, proximal weakness, presence of Beevor’s sign.
			18	4qB		
FSHD 7	female	22	4	4qA	14	typical facial weakness, scapular winging, proximal weakness, presence of Beevor’s sign.
			41	4qB		
FSHD 8	female	30	4	4qA	19	weakness of orbicularis oris muscle, scapular winging, arm abduction below shoulder level, weakness in hand and wrist extension, presence of Beevor’s sign, foot dorsiflexion weakness
			36	4qB		
FSHD 9	male	34	2	4qA	24	typical facial weakness, scapular winging, proximal weakness, arm abduction below shoulder level, lumbar lordosis
			13	4qA		
FSHD 10	male	38	5	4qA	17	typical facial weakness, scapular winging, arm abduction below shoulder level, weakness and atrophy of biceps asymmetrically, lumbar lordosis, presence of Beevor’s sign, foot dorsiflexion weakness
			19	4qB		
FSHD 11	male	35	3	4qA	25	facial involvement, proximal weakness, weakness in hand, presence of Beevor’s sign, foot dorsiflexion weakness.
			20	4qB		
FSHD 12	female	27	5	4qA	13	facial involvement, scapular winging, arm abduction below shoulder level, weakness in hand and wrist extension, presence of Beevor’s sign.
			56	4qA		

A total of 14 prenatal diagnostic procedures were performed following genetic counseling. Chorionic villus sampling (CVS) was conducated in 12 cases between 11 and 13^+ 6^ weeks of pregnancy, while amniocentesis was performed in 2 cases after 16 weeks of pregnancy (Table 2). OGM was used as the genetic diagnostic platform for the prenatal diagnosis of FSHD. The average mapping coveragewas ≥ 75X. Among the 14 pregnancies, 4 fetuses were found to have D4Z4 repeat counts ranging from 2 to 4 on the 4qA allele, resulting in pregnancy termination.

**Table 2:** Prenatal diagnosis results of 12 FSHD families

FSHD family	Maternal age	Gestational age	Sample type	OGM FSHD pipeline			Labeled enzyme
				Calculate repeat count	Haplotype	Coverage	
FSHD 1	31	11^+ 2^	chorionic villus	2	4qA	14	NLRS-Nb.BSSSI
				28	4qA	13	
FSHD 2	30	13^+ 5^	chorionic villus	3	4qA	77	DLS-DLE
				11	4qB	86	
FSHD 3 − 1	24	11^+ 4^	chorionic villus	4	4qA	28	DLS-DLE
				15	4qB	35	
FSHD 3 − 2	28	12^+ 5^	chorionic villus	15	4qB	65	DLS-DLE
				30	4qB	61	
FSHD 4 − 1	32	13^+ 2^	chorionic villus	3	4qA	14	NLRS-Nb.BSSSI
				22	4qB	52	
FSHD 4 − 2	33	12^+ 2^	chorionic villus	13	4qA	25	NLRS-Nb.BSSSI
				23	4qB	12	
FSHD 5	32	12^+ 5^	chorionic villus	31	4qA	62	DLS-DLE
				39	4qA	41	
FSHD 6	27	17^+ 4^	amniotic fluid	18	4qB	60	DLS-DLE
				37	4qB	35	
FSHD 7	22	11^+ 6^	chorionic villus	23	4qA	15	NLRS-Nb.BSSSI
				40	4qB	10	
FSHD 8	30	16^+ 1^	amniotic fluid	35	4qB	69	DLS-DLE
				43	4qB	29	
FSHD 9	33	12^+ 4^	chorionic villus	15	4qA	30	NLRS-Nb.BSSSI
				31	4qA	12	
FSHD 10	36	12^+ 5^	chorionic villus	19	4qB	78	DLS-DLE
				38	4qB	73	
FSHD 11	30	12^+ 5^	chorionic villus	17	4qA	80	DLS-DLE
				20	4qB	84	
FSHD 12	27	11^+ 3^	chorionic villus	52	4qA	25	DLS-DLE
				20	4qB	65	

#### Positive results of prenatal diagnosis of FSHD

**Fig. 3:**
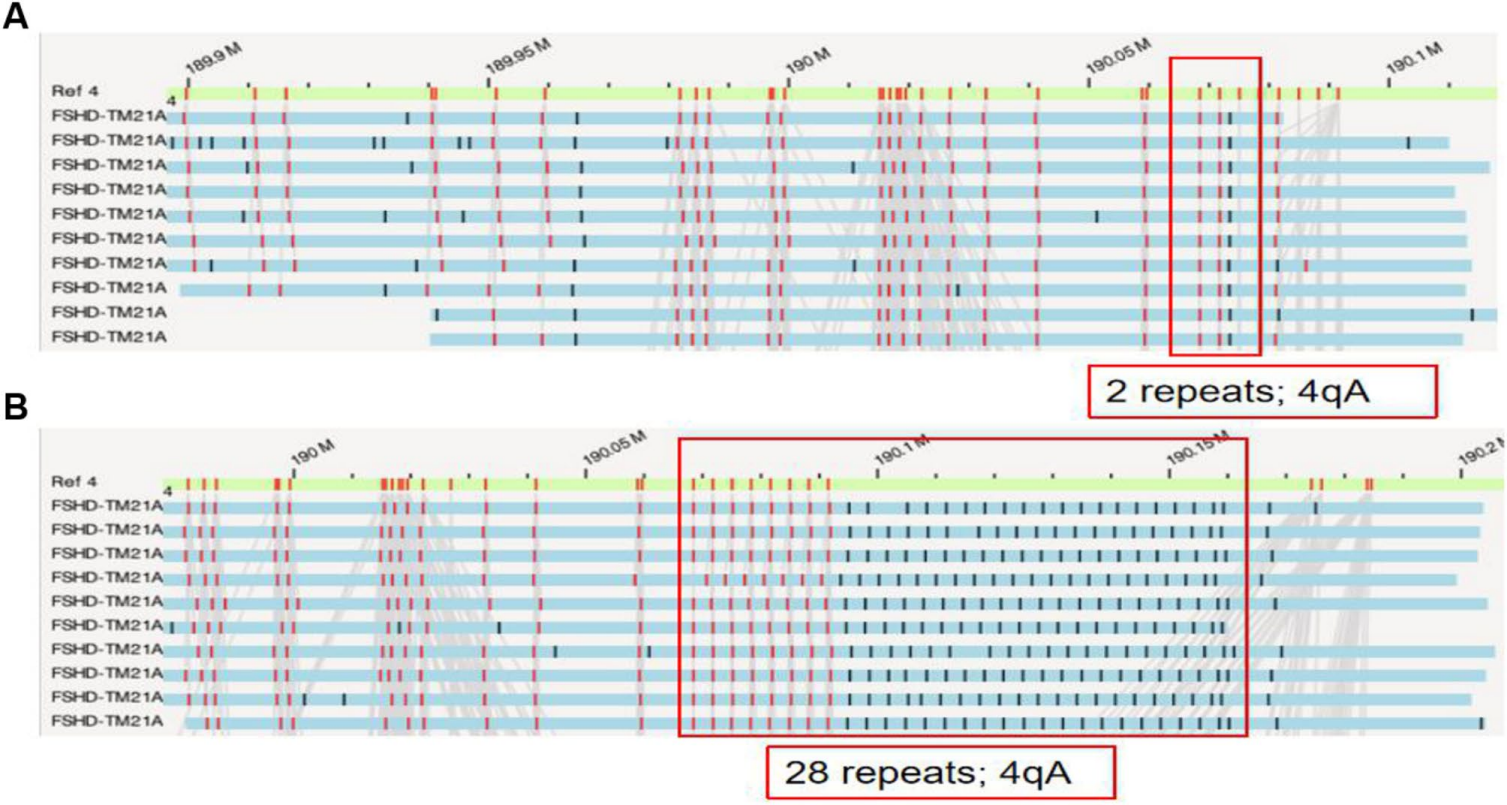
Prenatal diagnosis of the fetus in Family 1 with FSHD The reference is highlighted in green within the target region on chromosome 4q35 for comparison. The blue bars each show one optical map reading in the target region of the patient. (**A**) the fetus’s pathogenic chromosome 4, showing 2 D4Z4 repeats on the 4qA allele; (**B**) the fetus’s normal chromosome 4, showing 28 D4Z4 repeats on the 4qA allele

In Family 1, the proband had 2 D4Z4 repeats on the 4qA allele. Prenatal diagnosis was performed when the proband was 31 years old. OGM analysis revealed that the fetus inherited the same pathogenic chromosome, carrying 2 D4Z4 repeats on the 4qA allele (Fig. 3). The result indicated that the fetus was likely to be affected by FSHD. After genetic counselling, the couple decided to terminate the pregnancy.

**Fig. 4:**
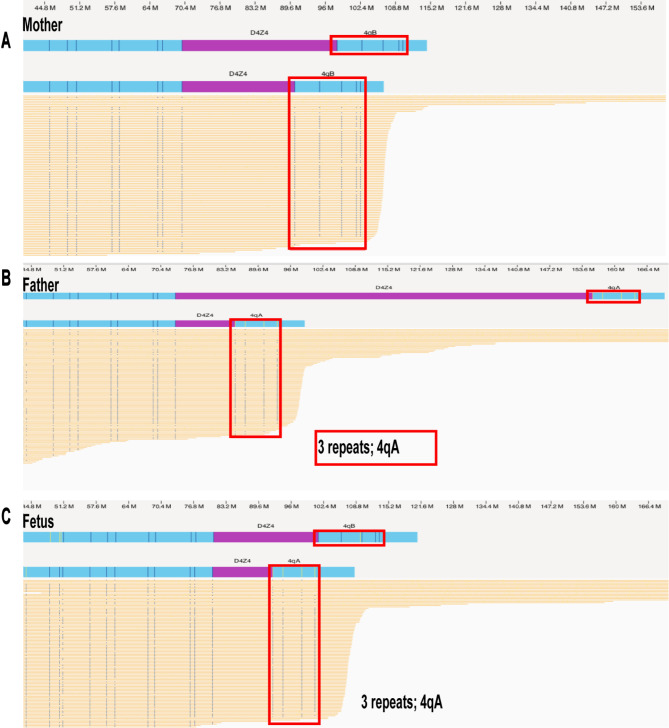
The OGM results of family 2 with FSHD The figure shows the OGM results for family 2, including the proband (father), mother, and fetus. The genetic analysis indicated that the fetus inherited the pathogenic 4qA allele with 3 D4Z4 repeats from the affected father

In Family 2, OGM was performed for both the couple and the fetus (Fig. 4). As shown in Fig. 4, the fetus inherited the pathogenic 4qA chromosome with 3 D4Z4 repeats from the father. For the other allele, the fetus inherited 11 D4Z4 repeats on the 4qB allele from the unaffected mother. Following genetic counseling, the pregnancy was terminated.

### Repeated prenatal diagnosis in Family 3 and family 4

**Fig. 5:**
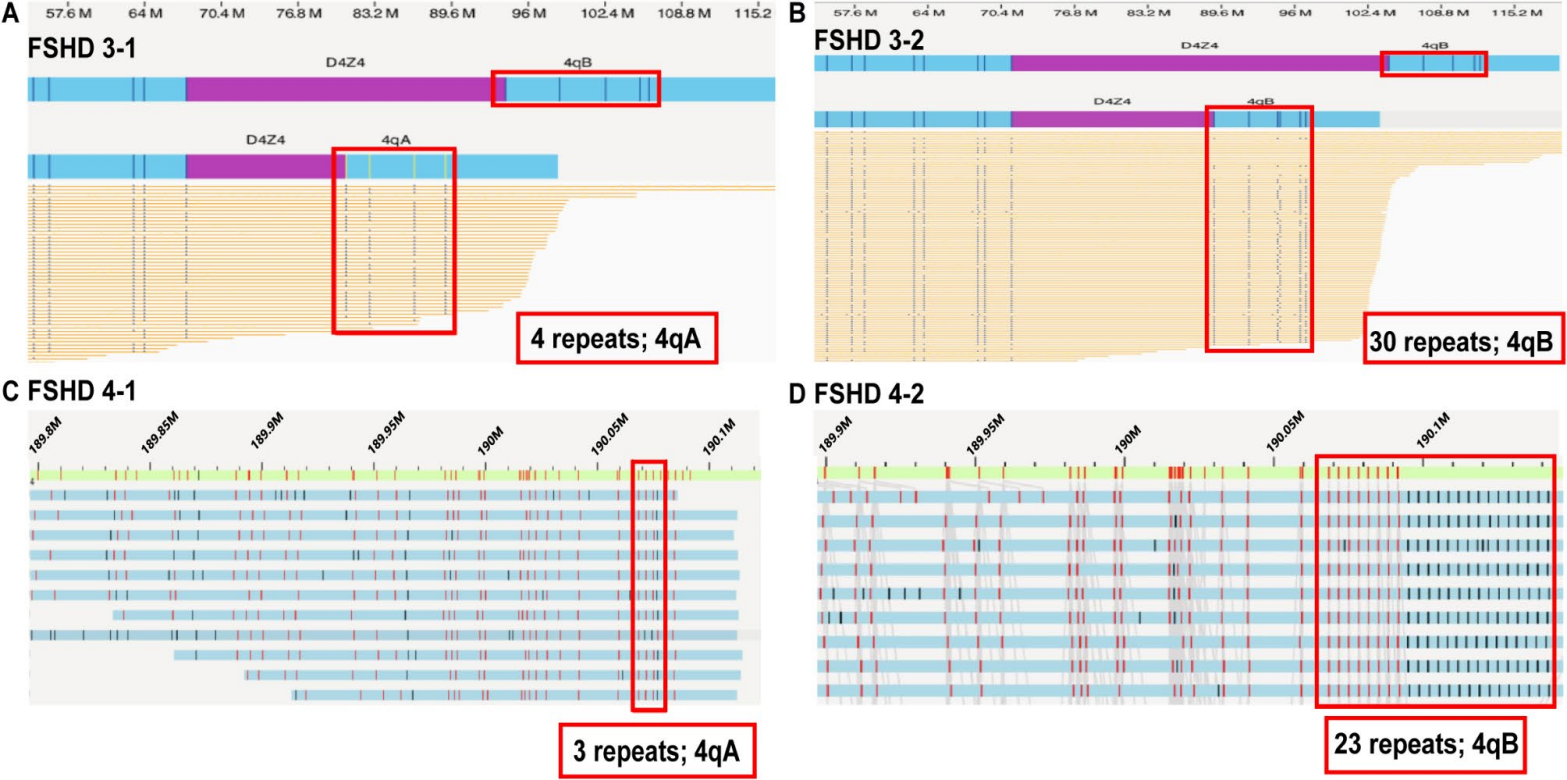
Two rounds of prenatal diagnosis in family 3 and 4 (**A**) The first fetus in Family 3 with 4 D4Z4 repeats on the 4qA allele. (**B**) The second fetus in Family 3 inherited the normal 30 D4Z4 repeats on 4qB allele from the affected mother. (**C**) The first fetus with 3 D4Z4 repeats on the 4qA allele in family 4. (**D**) The second fetus in Family 4 inherited the normal 23 D4Z4 repeats on 4qB allele from the affected proband

In Family 3 and Family 4, each family had two fetuses at different maternal ages, OGM tests had been conducted on two fetuses of each family. In Family 3, the first fetus (FSHD 3 − 1) inherited the pathogenic 4 D4Z4 repeats on the 4qA allele from the affected mother (Fig. 5A). Following genetic counselling, the pregnancy was terminated. The couple subsequently conceived again and sought a second prenatal diagnosis, hoping for a healthy fetus. The second fetus was healthy, inheriting the normal 30 D4Z4 repeats on the 4qB allele from the affected mother. The child was successfully delivered (Fig. 5B). During follow-up, the child continued to show normal development and remained in good health.

In Family 4, a similar situation occurred. The first prenatal diagnosis showed that the fetus had inherited the pathogenic 3 D4Z4 repeats on the 4qA allele from the affected father, resulting in pregnancy termination. In the second pregnancy, the fetus was found to inherit the normal 23 D4Z4 repeats on the 4qB allele from the affected proband, confirming a healthy outcome, and the child was delivered in good health.

## Discussion

Genetic analysis of FSHD is challenging due to the high degree of homology between the 4q35 and 10q26 regions, the variability in D4Z4 repeat numbers and the presence of somatic mosaicism [5, 12, 13]. The use of SB in prenatal diagnosis of FSHD is limited by its requirement for quality and quantity of DNA to have a molecular diagnosis [7, 14]. This often results in inadequate material for definitive results, necessitating repeated sampling—a significant challenge in prenatal setting due to associated risks and the time-sensitive nature of pregnancy. In 1999, Upadhyaya et al. conducted 12 prenatal diagnoses for FSHD families using SB and raised several practical issues related to its application [7]. Despite the long-established use of SB, recent reports on its use in prenatal diagnosis have been rare [15]. Prenatal diagnosis requires high accuracy and precision within strict time constraints. Literature review suggests that the use of SB in direct prenatal diagnosis has virtually declined in recent years, reflecting its impractical in this context. OGM has emerged as a transformative tool in genetic diagnostics, effectively addressing the shortcomings of SB [9, 10, 16]. OGM enables precise detection of D4Z4 repeat copy numbers and mosaicism—key factors in accurate FSHD diagnosis [10, 11]. These capabilities make OGM highly suitable for prenatal diagnosis, offering rapid and reliable results crucial for clinical care.

In this study, the analysis of D4Z4 repeat patterns in 100 unrelated healthy individuals from the Chinese Han population highlighted the value of OGM in examining D4Z4 repeats, further underscoring its diagnostic potential. Notably, three individuals carry 10 D4Z4 repeats on the 4qA allele but showed no clinical symptoms. Typically, the D4Z4 repeat array size in control individuals ranges from 11 to 100 units [17]. Later studies in European populations have reported the presence of 8–10 D4Z4 repeats on the 4qA allele in approximately 1–2% of individuals without clinical symptoms [18, 19]. In our study, the presence of 10 D4Z4 repeats on the 4qA allele was observed in 3.00% of the control Chinese Han population, suggesting that the cutoff for D4Z4 repeats may require further evaluation. We also detected one individual with 9 D4Z4 repeats on the 4qB allele. Lemmers et al. reported that this repeat count is frequently encountered in the normal population without apparent pathogenic consequences [20]. One case of mosaicism with a complex genotype was also observed in the control population. Mosaicism is common in FSHD patients [21], and our findings suggest that OGM is effective for detecting mosaicism. The frequency of 4qA and 4qB alleles in the control population is 38.80% and 61.20%, respectively, consistent with the findings of Attarian et al., who reported frequencies of 42% for 4qA and 58% for 4qB [22].

We performed 14 times prenatal diagnosis in 12 FSHD families. There were three types of samples in our study: peripheral blood, chorionic villi, and amniotic fluid. Blood samples are the easiest to process, as they can be directly used as DNA isolation input. In contrast, chorionic villus requires a minimum of 10 mg of tissue, which involves more complex and invasive procedures, while amniotic fluid requires cell culture, leading to a longer detection cycle. Regarding the methodology, DLS labeling offers several advantages for data acquisition. It does not enzymatically digest DNA, resulting in longer DNA strands suitable for analysis. Additionally, the analysis software includes a dedicated FSHD pipeline.

Our study observed a prenatal diagnostic rate of 28.57% for FSHD. The rate is quite low, given the 50% inheritance probability. This rate may reflect parental selection of healthy chromosomes during reproduction. Expanding the sample size in future research will enhance our understanding of FSHD prevalence and transmission patterns, providing a stronger basis for clinical guidelines. Two families (Family 3 and 4) underwent repeated prenatal testing, with OGM delivering accurate results that informed reproductive decisions.

Globally, OGM is gaining recognition as a mainstream genetic diagnostic method, particularly for conditions like FSHD [11, 23]. Unlike classic FSHD1, which is primarily associated with deletions of D4Z4 repeats on the 4qA allele, FSHD2 is characterized by digenic inheritance, requiring both a shortened 4qA allele and a mutation in the SMCHD1 gene. Given this complexity, the combination of OGM with next-generation sequencing (NGS) of *SMCHD1* holds great promise for diagnosing most cases of FSHD2 [24]. Dai et al. validated the accuracy and reliability of OGM in determining the number of D4Z4 repeats and in quantifying the level of mosaicism, comparing it with SB [9]. Our results further support OGM as a preferred method for prenatal diagnosis of FSHD1.

## Conclusions

In conclusion, OGM represents a significant advancement in prenatal diagnosis, overcoming the limitations of traditional methods like SB. By providing precise and timely results, OGM meets the rigorous demands of prenatal care, enabling families to make informed reproductive decisions.

## Data Availability

Datasets in this study are available from the corresponding author, upon reasonable requests.

## Abbreviations

FSHD: Facioscapulohumeral muscular dystrophy
SB: Southern blot
OGM: Optical genome mapping
CVS: Chorionic villus sampling
NGS: Next-generation sequencing
